# Bone tissue regeneration: role of osteocyte mechanosensing and mechanotransduction

**DOI:** 10.1093/stcltm/szag017

**Published:** 2026-04-15

**Authors:** Hadi Seddiqi, Jenneke Klein-Nulend, Jianfeng Jin

**Affiliations:** Department of Oral Cell Biology, Academic Centre for Dentistry Amsterdam (ACTA), University of Amsterdam and Vrije Universiteit Amsterdam, Amsterdam Movement Sciences, 1081 LA Amsterdam, The Netherlands; Department of Oral Cell Biology, Academic Centre for Dentistry Amsterdam (ACTA), University of Amsterdam and Vrije Universiteit Amsterdam, Amsterdam Movement Sciences, 1081 LA Amsterdam, The Netherlands; Department of Oral Cell Biology, Academic Centre for Dentistry Amsterdam (ACTA), University of Amsterdam and Vrije Universiteit Amsterdam, Amsterdam Movement Sciences, 1081 LA Amsterdam, The Netherlands

**Keywords:** bone, cell signaling, mesenchymal stem cells (MSCs), microenvironment, osteoblast, signal transduction, stem/progenitor, tissue regeneration

## Abstract

Critical-sized bone defects caused by trauma, tumor resection, injury, and/or surgical intervention are posing significant clinical challenges. Bone tissue regeneration is crucial for restoring critical-sized bone defects. Central to the bone regenerative capability is the dynamic interplay between bone cells, particularly osteocytes, which are the most abundant and long-lived bone cells, functioning as key mechanosensors in bone. Osteocytes detect mechanical stimuli, for example, fluid shear stress, compressive or tensile strain, and hydrostatic pressure, and convert these into biochemical signals through mechanotransduction. The biochemical signals (eg, calcium ions, Wnt, etc.) regulate osteoblast and osteoclast-mediated remodeling. Osteocytes communicate with osteoblasts and osteoclasts via paracrine factors, including nitric oxide, prostaglandins, and sclerostin. Moreover, estrogen deficiency is known to alter osteocyte mechanosensitivity, impair osteocyte signaling, and dysregulate bone remodeling. Understanding how mechanical and hormonal factors affect osteocyte signaling is essential for developing effective therapeutic interventions. This concise review explores the role of osteocyte mechanosensing and mechanotransduction in bone tissue regeneration to improve bone healing, especially in critical-sized bone defects. The cellular and molecular mechanisms underlying bone regeneration and remodeling are discussed, including the role of stem cells in bone regeneration, that is, osteogenic differentiation potential and secretion of bioactive factors that promote new bone formation and vascularization. Finally, we explore the translational and clinical implications of osteocyte mechanobiology, discussing current challenges and potential advancements in bone tissue engineering and regenerative medicine. By integrating fundamental mechanobiological principles with clinical strategies, this concise review highlights the clinical potential of modulating osteocyte behavior for improved bone regeneration.

## Graphical abstract

**Graphical Abstract:**
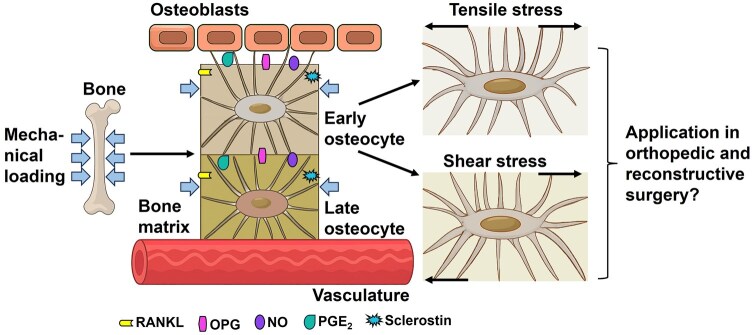

Significance statement Critical-sized bone defect treatment is a clinical challenge. This review explores how mechanical and hormonal factors affect osteocyte mechanosensing, which is crucial for developing effective clinical strategies for bone defect treatment. We cover recent advances in smart responsive biomaterials that interact with osteocytes and mimic mechanical signaling pathways, offering novel directions for enhancing bone tissue regeneration. We also discuss a surgical procedure for jawbone regeneration involving stem cells, underscoring the translational potential of integrating osteocyte mechanobiology with biomaterial and stem cell-based approaches to achieve faster and more reliable bone healing.

## Introduction

Bone has a remarkable capacity for self-repair. However, in many clinical scenarios, for example, critical-sized bone defects resulting from trauma, tumor resection, injury, severe vascular injuries/losses associated with bone damage, and/or surgical intervention, this natural regenerative ability is insufficient.^1^ The treatment of critical-sized bone defects remains a significant challenge in orthopedic and reconstructive surgery. Current clinical strategies, including autografts and allografts, are associated with limitations such as donor site morbidity, immunogenic responses, and poor integration, particularly in elderly or osteoporotic patients.^2^ Consequently, there is a growing need to develop novel biological and biomechanical strategies to accelerate bone repair and regeneration.

Bone regeneration is a physiological process involving inflammation, cell recruitment, matrix deposition, and remodeling.^3^ While osteoblasts and osteoclasts have long been recognized as the primary effectors of bone formation and resorption, osteocytes, the most abundant and long-lived bone cells (representing over 90% of the bone cell population during the resting phase), play a significant role as central coordinators of bone regeneration and remodeling.^4^ The osteocytes exert their coordinating role as part of the bone basic cellular system (BBCS), together with bone lining cells and stromal cells, forming a functional syncytium interconnected by gap junctions.^5^ Functioning as key mechanosensors, osteocytes initiate signaling cascades that regulate mechanotransduction processes.^6^ Through mechanotransduction, osteocytes translate mechanical stimuli, for example, fluid shear stress, compressive or tensile strain, and hydrostatic pressure, into biochemical signals.^7^^,^^8^ These biochemical signals regulate osteoclast and osteoblast activity during bone remodeling.^6^^,^^9^ Therefore, understanding the molecular mechanisms of osteocyte mechanosensing and mechanotransduction is vital for developing therapeutic strategies to boost bone regeneration.

This concise review aimed to address the so far largely unknown role of osteocytes in bone tissue regeneration, with a specific focus on their mechanosensing and mechanotransduction functions. First, we discuss the cellular and molecular mechanisms underlying bone regeneration and remodeling. Second, the specific osteocyte’s role in bone regeneration and remodeling is reviewed, emphasizing the osteocyte dendritic network, lacuno-canalicular system, and their involvement in regulating osteoblast and osteoclast activity as part of an interconnected cellular network, for example, the bone basic cellular system (BBCS). Third, we highlight how smart responsive biomaterials interact with osteocytes to mimic or enhance biological responses of osteocytes. Osteocyte mechanosensing and mechanotransduction within smart biomaterials are also reviewed. The clinical application of the mechanotransduction function of osteocytes is addressed through an analysis of the impact of osteocyte (dys)function. Finally, we explore osteocyte-informed bone tissue engineering strategies, including translational efforts such as a one-step surgical procedure for jawbone regeneration using adipose stem cells.

## Bone regeneration and remodeling

Bone regeneration and remodeling are essential processes that maintain skeletal integrity and facilitate bone healing in response to injury or physiological demands.^10^ Bone remodeling is a process whereby bone is constantly removed by osteoclasts through bone resorption and replaced with new bone formed by osteoblasts.^11^ Both bone resorption and formation are well coordinated by osteocytes in a temporal and spatial way to maintain skeletal integrity.^11^ The bone remodeling process is regulated by (i) activation, (ii) resorption, (iii) reversal, (iv) formation, and (v) termination.^11^ Each stage is characterized by specific cellular activities and biochemical interactions. However, sometimes the bone regenerative demand exceeds the normal potential for self-healing, such as in critical-sized bone defects (defect size length ≥1 cm, involving >50% of cortical diameter).^12^ Therefore, bone regeneration is needed to repair critical-sized bone defects, especially during the early healing phase, where vibrant changes in cellular and tissue composition alter the mechanical environment and therefore affect signalling pathways that orchestrate the healing process.^10^

## Osteocytes in bone regeneration and remodeling

Osteocytes coordinate osteoblast and osteoclast activity by secreting key signaling molecules, such as osteoprotegerin (OPG), sclerostin, dickkopf-related protein 1 (DKK1), prostaglandin E_2_ (PGE_2_), and nitric oxide (NO).^13^ Osteocytes regulate bone resorption by producing receptor activator of nuclear factor κB ligand (RANKL), which promotes osteoclast differentiation, and OPG, a decoy receptor for RANKL that inhibits osteoclastogenesis and thereby counterbalances bone resorption.^14^ In addition, osteocytes secrete sclerostin, which suppresses bone formation.^15^ These signals are modulated in response to mechanical loading, ensuring that bone adapts to functional demands, while also mediating between the body’s metabolic needs (eg, maintaining normal blood calcium levels) and the skeleton’s mechanical requirements.^7^^,^^16^ In osteocytes, the critical mechanosensitive ion channel piezo1 converts mechanical forces into biochemical signals via a Ca^2+^ influx, thereby regulating the Wnt/β-catenin, NF-κB, and yes-associated protein (YAP(/transcriptional co-activator with PDZ-binding motif (TAZ) pathways.^17^ Further elucidation of the role of mechanosensitive ion channels in bone regeneration is still needed, with a focus on targeted modulation of these ion channels in osteocytes in clinical settings, potentially through pharmacological agonists or gene-editing techniques, to enhance bone adaptation in patients with impaired mechanosensitivity.

Osteocytes detect mechanical loads using specialized mechanosensory structures.^7^^,^^8^ When bone is subjected to mechanical load, fluid flow through the lacuno-canalicular network generates shear stress on the osteocyte membranes, triggering intracellular signaling pathways. Osteocytes secrete several signaling molecules upon mechanical loading, that is, OPG, sclerostin, DKK1, PGE_2_, and NO.^13^ These signaling molecules modulate the activity of osteoclasts and/or osteoblasts to maintain bone integrity by stimulating bone resorption or new bone formation.^18^

Osteocyte apoptosis is another critical aspect of bone homeostasis.^19^ Apoptotic osteocytes can signal the recruitment of osteoclasts, initiating targeted bone resorption, though this is debated as apoptosis may sometimes be a consequence rather than a trigger of damage.^20^ For instance, blocking apoptosis with caspase inhibitors does not always prevent osteoclast activation or new cutting cone formation.^21^ Moreover, mechanical stimulation can induce RANKL expression independently of significant apoptosis via pathways like Wnt/β-catenin and PGE_2_.^22^ Thus, osteocyte apoptosis is one of several putative determinants in the multifactorial bone remodeling process. Modifications in the osteocyte lacunar morphology and perilacunar tissue properties can significantly alter local tissue deformation around lacunae, which may impact osteocyte mechanosensitivity.^20^ This process is essential during periods of high calcium demand, such as lactation, and contributes to maintaining matrix quality.^20^ Key signaling molecules secreted by osteocytes in response to mechanical loading are summarized in Table 1.

**Table 1.** Key signaling molecules involved in local regulation of bone regeneration and remodeling produced by osteocytes after mechanical (un)loading.

Molecule	Stimulus	Main signaling pathway affected	Function: regulatory outcome on bone remodeling	Reference(s)
**OPG**	Mech. loading	Wnt/β-catenin, RANKL	Inhibition of osteoclast differentiation	^13^ ^,^ ^14^
**Sclerostin**	Mech. loading / Mech. unloading	Wnt/β-catenin	Stimulation / inhibition of osteoblast differentiation and bone formation	^15^
**Dkk1**	Mech. unloading	Wnt/β-catenin	Inhibition of bone formation	^13^
**PGE_2_**	Mech. loading	NF-κB, YAP/TAZ	Increase in bone formation and mechanotransduction	^22^
**NO**	Mech. loading	cGMP–PKG	Stimulation of osteoblast activity; suppression of osteoclast formation	^13^
**RANKL**	Mech. unloading	RANK–NF-κB–NFATc1	Promotion of osteoclast differentiation and bone resorption	^14^ ^,^ ^22^

Abbreviations: DKK1, dickkopf-related protein 1; Mech. (un)loading, mechanical (un)loading; NO, nitric oxide; NOS, nitric oxide synthase; OPG, osteoprotegerin; PGE_2_, prostaglandin E_2_; RANKL, receptor activator of nuclear factor κB ligand; TAZ, transcriptional co-activator with PDZ-binding motif; YAP, yes-associated protein.

The capacity of osteocytes to regulate bone regeneration and remodeling is intimately dependent on the physicochemical environment conditions that support their survival, connectivity, and mechanosensing function.^9^ Osteocytes rely on specific physicochemical environment conditions, such as matrix stiffness, oxygen availability, pH balance, and ionic composition, to effectively detect and respond to mechanical stimuli.^23^ These physicochemical parameters not only influence osteocyte viability and morphology but also govern the organization of the lacuno-canalicular network, which is critical for cell–cell communication and the propagation of mechanosensing signals.^24^ Therefore, understanding how the osteocyte environment condition modulates mechanosensing and mechanotransduction provides essential insights into bone regeneration and remodeling. Moreover, the close interplay between osteocytes and their physicochemical environment highlights the environment as a potential therapeutic target to preserve bone quality and promote regeneration in clinical settings.^9^ The cellular environment is a three-dimensional (3D) structure consisting of extracellular matrix components, signaling molecules, and/or other cells.^9^ The osteocyte environment is defined by the lacuno-canalicular system that provides both mechanical and biochemical connectivity. Osteocytes experience constant exposure to mechanical and physicochemical conditions, including mechanical stress, nutrient and oxygen gradients, ionic composition, and extracellular pH within their environment.^9^ These mechanical forces and physicochemical environment conditions trigger complex intracellular pathways that regulate the release of mediators, which coordinate bone remodeling.^13^ Osteocytes are subjected to mechanical loads, causing deformations of the membrane, cytoskeleton, and/or nucleus, which elicit biochemical responses and secretion of signaling molecules into the environment.^9^ Both *in vivo* and *in vitro* studies remain essential to unravel the full complexity of osteocyte-environment interactions.

Engineering bone tissue through microenvironmental insights aims to replicate the native bone microenvironment, enabling precise regulation of cellular mechanotransduction, signaling, and extracellular matrix remodeling essential for bone regeneration.^9^ 3D-culture models and biomimetic scaffolds allow precise control of environment parameters, such as matrix viscoelasticity, to study osteocyte mechanobiology under both physiological and pathological conditions.^25^ For instance, polyacrylamide-azobenzene (PAMA) hydrogel with tunable stiffness (4-19 kPa) triggers immediate responses in mesenchymal stromal cells, including changes in cell shape and YAP localization.^26^ Reduced matrix stiffness promotes human stem cell proliferation, spreading, and osteogenic differentiation through YAP activation, and significantly enhances alveolar bone regeneration *in vivo*.^27^ Stimulation by fluid shear stress rapidly causes phosphorylation of several transforming growth factor-β (TGFβ) family R-Smads by enhancing multimerization and activation of several TGFβ and bone morphogenetic protein (BMP) type I receptors.^28^ Understanding and targeting these microenvironmental cues offers promising strategies for promoting bone regeneration, preserving osteocyte viability, and maintaining skeletal homeostasis in both healthy individuals and patients with trauma, osteoporosis, bone tumors, or other metabolic bone diseases.

## Bone tissue regeneration approaches

The regeneration of critical-sized bone defects, that is, those that cannot heal autonomously, remains a central challenge in orthopedic and reconstructive medicine. Traditional grafting strategies are limited by donor site morbidity, availability, and risk of immune rejection, which has accelerated the development of bone graft substitutes. The approaches using bone graft substitutes for bone tissue regeneration are (i) synthetic or natural 3D-scaffolds alone^29^; (ii) 3D-scaffolds coated by bioactive molecules^30^^,^^31^; (iii) 3D-scaffolds containing living cells^32^; (iv) 3D-scaffolds containing bioactive molecules and living cells^33^; and (v) smart responsive biomaterials.^34^ The use of smart responsive biomaterials in bone tissue regeneration approaches seems most beneficial, as they represent the most advanced approach by actively interacting with the cellular and mechanical environment to guide osteocyte function, enhance bone regeneration, and improve clinical outcomes.

Smart responsive biomaterials are designed to sense and react to environmental stimuli such as pH changes, enzymatic activity, ionic strength, temperature variation, ultrasound, magnetic fields, and/or mechanical loading.^34^ In the context of bone regeneration, the incorporation of mechanosensitive features is particularly critical, as bone is a highly dynamic tissue shaped by mechanical cues. Osteocyte-like cells, for example, differentiated mesenchymal stem cells or pre-osteocytes conditioned toward an osteogenic lineage, expressing markers like sclerostin and E11/gp38, have been integrated into 3D models of smart responsive biomaterials in an environment of living cells and/or mechanical loading.^35^ In bone tissue regeneration approaches, incorporation of smart responsive biomaterials with these osteocyte-like cells that mimic the physicochemical environment of osteocytes is used to enhance their mechanosensing and signaling functions and promote effective bone remodeling and repair.^34^

## Smart responsive biomaterials and osteocytes

Biomaterials that mimic the native environment of osteocytes can potentially establish “osteocyte-inspired” scaffolds capable of modulating cellular behavior in response to local mechanical forces. Incorporating osteocyte-informed principles into scaffold design may include the use of smart responsive biomaterials, that is, mechanosensitive nanoparticles, ion-channel activators, or responsive hydrogels, which release bioactive molecules upon mechanical and biochemical stimulation.^36^ Moreover, culture of osteocytes in dynamic culture systems provides a living model for testing and optimizing smart responsive biomaterials.^37^ Promotion of bone regeneration could be achieved by smart responsive biomaterials that interact with osteocytes, which dynamically modulate cellular signaling and mechanotransduction processes. For example, native cellulose scaffolds release osteogenic factors in response to hydrostatic pressure to allow new bone growth.^38^ Piezoelectric hydrogel (PiezoGEL) combined with gelatin methacryloyl (GelMA) enhances stem cell viability and osteogenic differentiation by upregulating RUNX2, COL1A1, and alkaline phosphatase (ALP) gene expression, thereby effectively reducing periodontal inflammation and increasing bone regeneration in mice.^39^

General osteogenic biomaterials, for example, basic PCL scaffolds, primarily provide structural support and promote broad osteogenesis without targeting specific osteocyte pathways.^40^ In contrast, true “osteocyte-informed” biomaterials are designed to modulate osteocyte-specific mechanosensing, such as Piezo1 activation leading to Ca^2+^ influx and sclerostin regulation.^22^ For instance, PiezoGEL mimics Piezo1-mediated signaling to upregulate osteocyte-specific genes, whereas generic PCL scaffolds lack this targeted response, highlighting limitations like insufficient *in vivo* evidence for direct osteocyte interaction. Future designs could incorporate Piezo1 ligands or sclerostin-inhibiting nanoparticles to strengthen the biomaterial-osteocyte link.^41^ Tailored properties, informed by patient-specific factors like age or disease-induced bone loss, enable personalized regenerative strategies that optimize clinical outcomes for fracture healing and metabolic bone diseases.

### Smart responsive biomaterials and osteocyte mechanosensing and mechanotransduction

**Figure 1.**
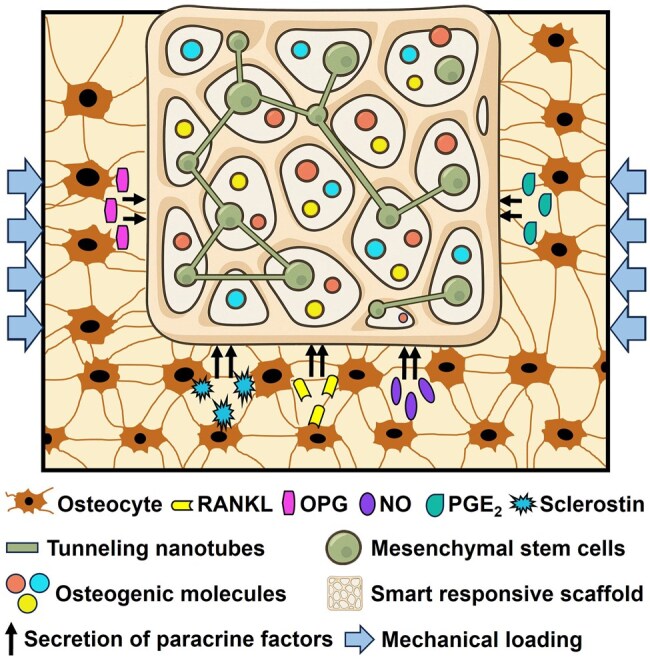
Innovative bone tissue engineering approach for bone defect repair. A construct containing stem cells and a scaffold in combination with surrounding bone enhances the biological functionality in critical-sized bone defect repair. This approach uses smart or responsive biomaterials, which mechanically interact with osteocyte networks, thereby stimulating osteogenic responses in a way that reflects the native bone environment. Osteocytes in the surrounding host bone secrete signaling molecules, for example, RANKL, OPG, NO, PGE_2_, and sclerostin, which affect bone regeneration and repair, in response to physiological mechanical loading. NO, nitric oxide; OPG, osteoprotegerin; PGE_2_, prostaglandin E_2_; RANKL, receptor activator of nuclear factor κB ligand.

Smart responsive biomaterials designed for bone tissue engineering aim to mimic or modulate the osteocyte mechanotransduction process by coupling environmental cues with controlled bioactivity.^42^ Such biomaterials not only provide structural support but also mechanically interact with osteocyte networks, thereby stimulating osteogenic responses in a way that reflects the native bone environment, often involving the differentiation of integrated mesenchymal stem cells toward osteocyte-like phenotypes (Figure 1). For instance, 3D-printed polycaprolactone (PCL) scaffolds reinforced by ceramic-based piezoelectric material, generating local electrical stimulation under mechanical stress or generating a mechanical response under external electrical stimulation, directly improve osteogenic differentiation.^43^ Similarly, soft hydrogels (stiffness: ∼0.5 kPa) mimicking soft bone marrow stiffness functionalized with mechanosensitive linkers, that is, matrix metalloproteinase-cleavable sites and RGD-based adhesive sites, enhance spreading and proliferation of encapsulated myoblasts,^44^ which have been shown to produce sclerostin as a myokine, potentially contributing to muscle-bone crosstalk in addition to bone-derived sclerostin.^45^ The myoblast-containing soft hydrogels cultured on rigid surfaces, mirroring the microenvironment of bone defects *in vivo*, show cell migration toward the interface and osteogenic differentiation in the presence of bone morphogenetic protein-2 (BMP2).^44^ The smart responsive biomaterials not only provide structural support but also dynamically interact with the osteocyte network to promote bone formation in a manner that closely reflects the native bone environment (Figure 1). Several factors affect the communication between osteocytes and the native/engineering environment, for example, extracellular matrix composition, gap junctions, hormones, aging, tunneling nanotubes, and mechanotransduction cascades. Below special attention will be given to tunneling nanotubes and mechanotransduction cascades, since these two factors play pivotal roles in bridging direct cellular interconnectivity and dynamic mechanical signaling, offering innovative targets for enhancing osteocyte-mediated bone regeneration in clinical tissue engineering applications.

### Tunneling nanotubes

Tunneling nanotubes are actin-rich cytoplasmic extensions (diameter 50-200 nm) that form direct physical and functional connections between osteocytes and neighboring cells.^40^ These transient, membrane-bound structures fascinate avenues of intercellular communication.^40^ For instance, tunneling nanotubes mediate the exchange of organelles (eg, mitochondria, lysosomes), plasma membrane components (eg, lipids, receptors), and cytoplasmic molecules (eg, calcium ions, microRNAs) of pre-osteoblasts.^46^ Moreover, via tunneling nanotubes, mitochondria are transmitted by osteocytes into adjacent cells to communicate with them, which supports energy demands and enhances cellular survival under stress conditions, such as hypoxia or mechanical strain. This has been observed primarily in *in vitro* models, as *in vivo* osteocytes are enclosed in lacunae, potentially relying instead on extracellular vesicles of mitochondrial origin or associated molecules for similar functions.^47^ Tunneling nanotube-mediated communication is critical in coordinating bone remodeling processes, particularly during stress responses, where osteocytes use tunneling nanotubes to transmit survival signals, such as anti-apoptotic proteins, or initiate programmed cell apoptosis by transferring pro-apoptotic signals.^48^ This dynamic signaling regulates the balance between bone formation and resorption, ensuring adaptive bone homeostasis in response to environmental challenges.

Incorporating tunneling nanotube-inspired mechanisms into biomaterial design enables enhanced intercellular signaling within 3D-scaffolds.^49^ Nanostructured fibrous scaffolds with interconnected porosity promote the formation of tunneling nanotube-like connections between cells, supporting synchronized mechanosensitivity across cell-seeded scaffolds.^49^ Furthermore, responsive biomaterials could be used to facilitate or stabilize tunneling nanotube formation under mechanical loading, ensuring efficient signal propagation and integration with host bone tissue. This tunneling nanotube-mimicking approach holds promise for improving both the efficiency and fidelity of cellular communication in bone regeneration strategies.

### Mechanotransduction cascades

The mechanotransduction cascades, that is, mechanocoupling, biochemical coupling, transmission of biochemical signals, and effector cell response in osteocytes, are a complex regulatory process between cells and their environment.^50^ Mechanotransduction in osteocytes involves the conversion of mechanical stimuli into biochemical signals that regulate bone formation and/or resorption. Smart responsive biomaterials can interfere with mechanotransduction cascades by embedding mechanosensitive components that release osteoinductive factors in response to mechanical strain.^35^ For instance, mechanically activated microcapsules have been fabricated to deliver specific drugs in response to a mechanically loaded environment for the smart regeneration of musculoskeletal tissues.^51^ These approaches leverage the osteocyte’s inherent mechanotransduction machinery, using it as a biological blueprint for scaffold design.

By integrating tunneling nanotube-inspired intercellular communication with the orchestration of osteocyte mechanotransduction cascades, next-generation smart responsive biomaterials can achieve more precise, adaptive, and physiologically relevant control over bone regeneration and remodeling. These approaches move beyond passive support to actively harness osteocyte biology, offering transformative potential for clinical translation.

## Clinical impact of osteocyte (dys)function

Appropriate osteocyte function maintains bone remodeling and mineral homeostasis, while osteocyte dysfunction impairs signaling pathways, which contribute to skeletal disorders such as osteoporosis, osteoarthritis, and impaired bone regeneration.^52^ Notably, estrogen deficiency changes the osteocyte’s cytoskeleton, primary cilium, and calcium signaling pathways, which disrupts balanced bone remodeling.^53^ Deleterious effects of estrogen depletion on skeletal mechanical adaptation appear at the level of mechanosensation; that is, osteocytes lose the ability to sense small (physiological) mechanical stimuli.^54^ Clinically, this manifests as delayed fracture bone healing, greater susceptibility to non-union, and heightened fracture risk in osteoporotic patients.^55^

One of the most well-characterized consequences of impaired osteocyte signaling is the overexpression of sclerostin, a potent inhibitor of the Wnt/β-catenin pathway.^15^ Elevated sclerostin levels suppress osteoblast activity and bone formation,^56^ exacerbating bone loss in estrogen-deficient states.^57^ This knowledge has led to the development of anti-sclerostin antibodies, such as romosozumab, which restore bone mass and reduce fracture incidence.^58^ Furthermore, osteocyte markers, especially OPG and sclerostin, play a critical role in classifying subjects with osteoporosis.^59^ Together, these insights highlight the translational importance of osteocyte biology, positioning osteocyte dysfunction not only as a mechanistic driver of, for example, osteoporosis, but also as a promising target for diagnostic and therapeutic innovations.

### Osteocyte orientation

The spatial orientation of osteocytes within the bone matrix plays a critical role in determining the efficiency of mechanosensing and subsequent mechanotransduction.^60^ Osteocyte morphology and orientation seem to be affected by the mechanical loading direction.^4^^,^^61–63^ Round osteocytes are much more mechanosensitive than elongated cells.^64^ It is unknown whether local osteocyte shape in jawbone may affect local bone mass. Properly aligned osteocytes, with dendritic processes extending along the principal loading directions, optimize the detection of fluid shear stresses and mechanical strains.^65^ Disruption of this orientation, as observed in osteoporotic bone, aging, or certain metabolic conditions, compromises the osteocyte network’s ability to coordinate bone remodeling.^66^ Clinically, altered orientation has been associated with impaired fracture healing, reduced adaptive responses to mechanical loading, and increased skeletal fragility.^67^ The differences in 3D morphology of osteocytes and their lacunae in long bones of different pathologies with different bone mineral density reflect an adaptation to matrix strain due to different external loading conditions.^68^ Since direct mechanosensing of matrix strain likely occurs by the cell bodies, the differences in osteocyte morphology and their lacunae indicate differences in osteocyte mechanosensitivity.^68^ The exact relationship between osteocyte morphology, bone architecture, and the type of osteogenesis with which they were formed, for example, static osteogenesis in primary woven bone leading to globular, disordered osteocytes, versus dynamic osteogenesis in secondary lamellar bone resulting in orderly ellipsoidal arrangements, is very complex and to be explored further.^69^ Thus, osteocyte orientation not only is a determinant of bone strength but also a potential diagnostic marker of bone dysfunction. From a therapeutic perspective, interventions that preserve or restore osteocyte alignment, such as mechanical loading regimens, biomaterial scaffolds designed to guide cellular orientation, or pharmacological strategies targeting cytoskeletal dynamics, may offer promising avenues to enhance bone repair and regeneration in clinical settings.

### One-step surgical procedure: clinical studies on jawbone regeneration

Long-term safety of adipose stem cell supplementation in combination with calcium phosphate ceramics for jawbone reconstruction has been shown.^70^ Adipose stem cell supplementation enhances bone regeneration in the short term,^71^ and does not lead to abnormalities, clinically and radiologically, in the long term.^70^ This warrants further studies to evaluate whether, for example, using higher dosages of adipose stem cells, altered scaffold properties, or application of other adipose tissue processing methods may enhance bone formation without causing side effects.^70^ These studies open new possibilities for a variety of cell-based bone tissue engineering applications, including osteocyte-informed bone tissue engineering.

## Osteocyte-informed bone tissue engineering strategies

Advances in bone tissue engineering increasingly emphasize the integration of osteocyte biology to enhance regenerative outcomes. Cell-seeded 3D scaffolds play a central role in this approach, with materials engineered to replicate the native lacuno-canalicular network that enables osteocyte signaling and load transmission.^72^ Optimized mechanical properties and anisotropy allow scaffolds to transmit physiological mechanical strains, thereby fostering cell–cell communication and promoting mechanotransduction.^29^ Such designs aim to create an environment where osteocytes can sense and respond to mechanical cues in a manner similar to native bone.

Bioreactor systems have further advanced the field by enabling dynamic culture conditions, that is, fluid flow, that mimic *in vivo* mechanical environments.^73^^,^^74^ Pulsating fluid flow creates strain patterns, thereby stimulating osteocytes to release anabolic factors such as prostaglandins, NO, and insulin-like growth factor-1 (IGF1).^75^^,^^76^ A single bout of pulsating fluid flow with indirect associated release of biochemical factors stimulates osteoblast differentiation in the long-term.^77^ Incorporating mechanosensitive cues directly into biomaterials offers additional opportunities to activate osteocyte-related pathways without external devices.^78^ These biomaterials generate biochemical signals in response to loading, thereby reinforcing osteogenic activity.

Differentiation of MSCs into osteocyte-like phenotypes, or genetic modulation of osteogenic cells to enhance mechanosensitivity, allows the creation of constructs that more closely recapitulate the osteocyte’s regulatory role in bone remodeling.^79^ Preclinical studies have demonstrated that scaffolds with osteocyte-mimetic cues or seeded with osteocyte-like cells enhance new bone formation, vascularization, and integration of scaffolds into a bone defect.^80^ Looking forward, combining osteocyte-informed scaffolds, dynamic bioreactor conditioning, and novel biomaterials offers a promising pathway toward personalized, clinically translatable solutions for critical-sized bone defect repair and skeletal regeneration.

The development of smart responsive biomaterials capable of adaptive feedback, that is, sensing mechanical loading and modulating cellular behavior accordingly, represents a promising frontier. *In vitro* and *in vivo* models that accurately replicate the osteocyte lacuno-canalicular system and dynamic mechanical environment—considering variations in arrangement based on the context of formation (for example, static vs. dynamic osteogenesis), bone structure (trabecular vs. compact), and architecture of the bone to be replaced—are essential to elucidate how mechanical and biochemical signals interact to regulate bone remodeling.

## Future directions

Advancing the field of bone tissue regeneration requires a multifaceted approach that builds on the foundational understanding of osteocyte mechanosensing and mechanotransduction.

Supplementation with adipose stem cells enhances bone formation and vascularization, offering a clinically viable alternative to autologous grafts. Future efforts should focus on integrating adipose stem cells with smart responsive scaffolds that enable controlled osteogenic and angiogenic responses. The demonstrated efficacy of adipose stem cell-supplemented calcium phosphate bone substitutes in jawbone regeneration indicates readiness for orthopedic translation, particularly for treating critical-sized bone defects, non-union fractures, and osteoporotic bone loss. Combining adipose stem cell-based strategies with advances in mechanically responsive biomaterials and osteocyte-targeted mechanobiology may further improve tissue integration and functional recovery, paving the way for next-generation, patient-specific regenerative therapies in orthopedic and reconstructive medicine. Forward-looking research directions include (1) developing AI-driven models to predict osteocyte responses in patient-specific environments; (2) exploring hybrid biomaterials with real-time feedback sensors for *in vivo* monitoring of osteocyte signaling; (3) conducting large-scale clinical trials on adipose stem cell-supplemented smart biomaterials for orthopedic applications beyond jaw regeneration; and (4) investigating the impact of aging and comorbidities, for example, diabetes, on osteocyte mechanobiology to tailor therapies. Addressing translational challenges, including scalability, regulatory approval, and long-term safety, will be essential for clinical implementation.

## Conclusions

**Figure 2.**
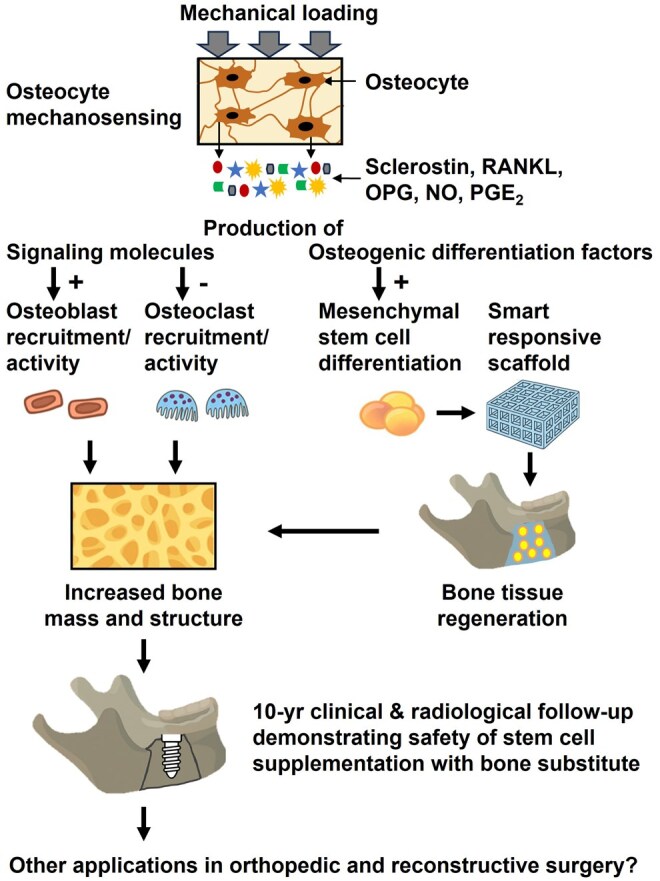
Schematic overview of the role of osteocytes in bone tissue regeneration. Mechanical loading stimulates the production of several signaling molecules, such as RANKL, OPG, NO, PGE_2_, and sclerostin by osteocytes. These signaling molecules not only regulate bone homeostasis but also affect the osteogenic differentiation of stem cells. NO, nitric oxide; OPG, osteoprotegerin; PGE_2_, prostaglandin E_2_; RANKL, receptor activator of nuclear factor κB ligand.

Osteocytes serve as pivotal mechanosensors in bone tissue regeneration, orchestrating remodeling through intricate mechanotransduction pathways that translate mechanical stimuli into biochemical signals regulating osteoblast and osteoclast activity (Figure 2). The lacuno-canalicular network and paracrine factors enable precise coordination of bone homeostasis. The integration of osteocytes and smart responsive biomaterials that mimic the osteocyte environment condition allows the development of new bone tissue engineering strategies with transformative potential. With proven safety and long-term efficacy in jawbone regeneration, adipose stem cell-supplemented calcium phosphate substitutes are now poised for orthopedic implementation, particularly in the treatment of critical-sized bone defects, delayed unions, and osteoporotic fractures. Future clinical research should prioritize standardized adipose stem cell processing protocols, patient selection criteria, and outcome measures to ensure reproducibility and regulatory acceptance. This will underscore the clinical implications of osteocyte mechanosensing and mechanotransduction, enhancing disease management across prognosis, treatment, prevention, and diagnosis.

## Data Availability

There are no new data associated with this article.

## References

[1] Migliorini F , La Padula G, Torsiello E, Spiezia F, Oliva F, Maffulli N. Strategies for large bone defect reconstruction after trauma, infections or tumour excision: a comprehensive review of the literature. Eur J Med Res. 2021;26:118. 10.1186/s40001-021-00593-9 34600573 PMC8487570

[2] Baldwin P , Li DJ, Auston DA, Mir HS, Yoon RS, Koval KJ. Autograft, allograft, and bone graft substitutes: clinical evidence and indications for use in the setting of orthopaedic trauma surgery. J Orthop Trauma. 2019;33:203-213. 10.1097/bot.0000000000001420 30633080

[3] Donos N , Akcali A, Padhye N, Sculean A, Calciolari E. Bone regeneration in implant dentistry: Which are the factors affecting the clinical outcome? Periodontol 2000. 2023;93:26-55. 10.1111/prd.12518 37615306

[4] Cao W , Helder MN, Bravenboer N, et al. Is there a governing role of osteocytes in bone tissue regeneration? Curr Osteoporos Rep. 2020;18:541-550. 10.1007/s11914-020-00610-6 32676786 PMC7532966

[5] Palumbo C , Ferretti M. The osteocyte: from “prisoner” to “orchestrator”. JFMK. 2021;6:28. 10.3390/jfmk6010028 33802907 PMC8006231

[6] Burger EH , Klein‐Nulend J. Mechanotransduction in bone—role of the lacunocanalicular network. Faseb J. 1999;13:S101-S112. 10.1096/fasebj.13.9001.s101 10352151

[7] Klein-Nulend J , Bakker AD, Bacabac RG, Vatsa A, Weinbaum S. Mechanosensation and transduction in osteocytes. Bone. 2013;54:182-190. 10.1016/j.bone.2012.10.013 23085083

[8] Klein‐Nulend J , van der Plas A, Semeins CM, et al. Sensitivity of osteocytes to biomechanical stress in vitro. Faseb J. 1995;9:441-445. 10.1096/fasebj.9.5.7896017 7896017

[9] Jin J , Bakker AD, Wu G, Klein-Nulend J, Jaspers RT. Physicochemical niche conditions and mechanosensing by osteocytes and myocytes. Curr Osteoporos Rep. 2019;17:235-249. 10.1007/s11914-019-00522-0 31428977 PMC6817749

[10] Duda GN , Geissler S, Checa S, Tsitsilonis S, Petersen A, Schmidt-Bleek K. The decisive early phase of bone regeneration. Nat Rev Rheumatol. 2023;19:78-95. 10.1038/s41584-022-00887-0 36624263

[11] Bolamperti S , Villa I, Rubinacci A. Bone remodeling: an operational process ensuring survival and bone mechanical competence. Bone Res. 2022;10:48. 10.1038/s41413-022-00219-8 35851054 PMC9293977

[12] Sanders DW , Bhandari M, Guyatt G, et al.; SPRINT Investigators S. Critical-sized defect in the tibia: is it critical? Results from the SPRINT trial. J Orthop Trauma. 2014;28:632-635. 10.1097/bot.0000000000000194 25233157

[13] Choi JUA , Kijas AW, Lauko J, Rowan AE. The mechanosensory role of osteocytes and implications for bone health and disease states. Front Cell Dev Biol. 2021;9:770143. 10.3389/fcell.2021.770143 35265628 PMC8900535

[14] Udagawa N , Koide M, Nakamura M, et al. Osteoclast differentiation by RANKL and OPG signaling pathways. J Bone Miner Metab. 2021;39:19-26. 10.1007/s00774-020-01162-6 33079279

[15] Marini F , Giusti F, Palmini G, Brandi ML. Role of wnt signaling and sclerostin in bone and as therapeutic targets in skeletal disorders. Osteoporos Int. 2023;34:213-238. 10.1007/s00198-022-06523-7 35982318

[16] Rubinacci A , Covini M, Bisogni C, et al. Bone as an ion exchange system: evidence for a link between mechanotransduction and metabolic needs. Am J Physiol Endocrinol Metab. 2002;282:E851-E864. 10.1152/ajpendo.00367.2001 11882505

[17] Zhang Y , Su S-a, Li W, et al. Piezo1-mediated mechanotransduction promotes cardiac hypertrophy by impairing calcium homeostasis to activate calpain/calcineurin signaling. Hypertension. 2021;78:647-660. 10.1161/hypertensionaha.121.17177 34333987

[18] Seddiqi H , Klein-Nulend J, Jin J. Osteocyte mechanotransduction in orthodontic tooth movement. Curr Osteoporos Rep. 2023;21:731-742. 10.1007/s11914-023-00826-2 37792246 PMC10724326

[19] Ru JY , Wang YF. Osteocyte apoptosis: the roles and key molecular mechanisms in resorption-related bone diseases. Cell Death Dis. 2020;11:846. 10.1038/s41419-020-03059-8 33046704 PMC7552426

[20] Sang W , Ural A. Quantifying how altered lacunar morphology and perilacunar tissue properties influence local mechanical environment of osteocyte lacunae using finite element modeling. J Mech Behav Biomed Mater. 2022;135:105433. 10.1016/j.jmbbm.2022.105433 36099785

[21] Cardoso L , Herman BC, Verborgt O, Laudier D, Majeska RJ, Schaffler MB. Osteocyte apoptosis controls activation of intracortical resorption in response to bone fatigue. J Bone Miner Res. 2009;24:597-605. 10.1359/jbmr.081210 19049324 PMC2659511

[22] Plotkin LI , Gortazar AR, Davis HM, et al. Inhibition of osteocyte apoptosis prevents the increase in osteocytic receptor activator of nuclear factor κB ligand (RANKL) but does not stop bone resorption or the loss of bone induced by unloading. J Biol Chem. 2015;290:18934-18942. 10.1074/jbc.M115.642090 26085098 PMC4521013

[23] Sharma D , Larriera AI, Palacio-Mancheno PE, et al. The effects of estrogen deficiency on cortical bone microporosity and mineralization. Bone. 2018;110:1-10. 10.1016/j.bone.2018.01.019 29357314 PMC6377161

[24] Sang W , Ural A. Influence of osteocyte lacunar-canalicular morphology and network architecture on osteocyte mechanosensitivity. Curr Osteoporos Rep. 2023;21:401-413. 10.1007/s11914-023-00792-9 37273086

[25] Bernero M , Zauchner D, Müller R, Qin XH. Interpenetrating network hydrogels for studying the role of matrix viscoelasticity in 3D osteocyte morphogenesis. Biomater Sci. 2024;12:919-932. 10.1039/d3bm01781h 38231154 PMC10863643

[26] Ansari A , Bhowmik S, Zhang K, et al. A visible light‐responsive hydrogel to study the effect of dynamic tissue stiffness on cellular mechanosensing. Adv Funct Materials. 2025;35:2501585. 10.1002/adfm.202501585

[27] Wu S , Chai Z, Yang Y, et al. Effect of matrix stiffness on the osteogenic differentiation of human periodontal ligament stem cells in a three-dimensional culture hydrogel: a preliminary study. ACS Biomater Sci Eng. 2025;11:5616-5626. 10.1021/acsbiomaterials.5c01151 40856628

[28] Monteiro DA , Dole NS, Campos JL, et al. Fluid shear stress generates a unique signaling response by activating multiple TGFβ family type I receptors in osteocytes. Faseb J. 2021;35:e21263. 10.1096/fj.202001998r 33570811 PMC7888383

[29] Zamani Y , Amoabediny G, Mohammadi J, et al. 3D-printed poly (Ɛ-caprolactone) scaffold with gradient mechanical properties according to force distribution in the mandible for mandibular bone tissue engineering. J Mech Behav Biomed Mater. 2020;104:103638. 10.1016/j.jmbbm.2020.103638 32174396

[30] Seddiqi H , Abbasi-Ravasjani S, Moghaddaszadeh A, et al. Osteogenic differentiation by MC3T3-E1 pre-osteoblasts is enhanced more on wet-chemically surface-modified 3D-printed poly-e-caprolactone scaffolds than on plasma-assisted modified scaffolds. Appl Surf Sci. 2024;671:160750. 10.1016/j.apsusc.2024.160750

[31] Moghaddaszadeh A , Ghiasvand ME, Seddiqi H, Abbasi-Ravasjani S, Klein-Nulend J. Osteogenic differentiation by pre-osteoblasts is enhanced more on 3D-Printed poly-ɛ-caprolactone scaffolds coated with collagen and hydroxyapatite than on poly-ɛ-caprolactone/hydroxyapatite composite scaffolds coated with collagen. J Biomater Appl. 2025;0:8853282251392820-8853282251392815. 10.1177/08853282251392820 PMC12957415 41151772

[32] Zamani Y , Mohammadi J, Amoabediny G, Helder MN, Zandieh-Doulabi B, Klein-Nulend J. Bioprinting of alginate-encapsulated pre-osteoblasts in PLGA/β-TCP scaffolds enhances cell retention but impairs osteogenic differentiation compared to cell seeding after 3D-printing. Regen Eng Transl Med. 2021;7:485-493. 10.1007/s40883-020-00163-1

[33] Moghaddaszadeh A , Seddiqi H, Najmoddin N, Abbasi-Ravasjani S, Klein-Nulend J. Biomimetic 3D-printed PCL scaffold containing a high concentration carbonated-nanohydroxyapatite with immobilized-collagen for bone tissue engineering: enhanced bioactivity and physicomechanical characteristics. Biomed Mater. 2021;16:065029. 10.1088/1748-605x/ac3147 34670200

[34] Yuan X , Zhu W, Yang Z, et al. Recent advances in 3D printing of smart scaffolds for bone tissue engineering and regeneration. Adv Mater. 2024;36:2403641. 10.1002/adma.202403641 38861754

[35] Wei H , Cui J, Lin K, Xie J, Wang X. Recent advances in smart stimuli-responsive biomaterials for bone therapeutics and regeneration. Bone Res. 2022;10:17. 10.1038/s41413-021-00180-y 35197462 PMC8866424

[36] Delint RC , Jaffery H, Ishak MI, Nobbs AH, Su B, Dalby MJ. Mechanotransducive surfaces for enhanced cell osteogenesis, a review. Biomater Adv. 2024;160:213861. 10.1016/j.bioadv.2024.213861 38663159

[37] Lipreri MV , Di Pompo G, Boanini E, et al. Bone on-a-chip: a 3D dendritic network in a screening platform for osteocyte-targeted drugs. Biofabrication. 2023;15:045019. 10.1088/1758-5090/acee23 37552982

[38] Latour ML , Pelling AE. Mechanosensitive osteogenesis on native cellulose scaffolds for bone tissue engineering. J Biomech. 2022;135:111030. 10.1016/j.jbiomech.2022.111030 35288315

[39] Roldan L , Montoya C, Solanki V, et al. A novel injectable piezoelectric hydrogel for periodontal disease treatment. ACS Appl Mater Interfaces. 2023;15:43441-43454. 10.1021/acsami.3c08336 37672788

[40] Zhang JQ , Takahashi A, Gu JY, et al. In vitro and in vivo detection of tunneling nanotubes in normal and pathological osteoclastogenesis involving osteoclast fusion. Lab Invest. 2021;101:1571-1584. 10.1038/s41374-021-00656-9 34537825

[41] Qin L , Liu W, Cao H, Xiao G. Molecular mechanosensors in osteocytes. Bone Res. 2020;8:23. 10.1038/s41413-020-0099-y 32550039 PMC7280204

[42] Khan HM , Liao X, Sheikh BA, et al. Smart biomaterials and their potential applications in tissue engineering. J Mater Chem B. 2022;10:6859-6895. 10.1039/d2tb01106a 36069198

[43] Meng D , Hou Y, Zubairi H, et al. Ceramic-based piezoelectric material reinforced 3D printed polycaprolactone bone tissue engineering scaffolds. Mater Des. 2025;257:114542. 10.1016/j.matdes.2025.114542

[44] Wei Q , Young J, Holle A, et al. Soft hydrogels for balancing cell proliferation and differentiation. ACS Biomater Sci Eng. 2020;6:4687-4701. 10.1021/acsbiomaterials.0c00854 33455192

[45] Magarò MS , Bertacchini J, Florio F, et al. Identification of sclerostin as a putative new myokine involved in the muscle-to-bone crosstalk. Biomedicines. 2021;9:71. 10.3390/biomedicines9010071 33445754 PMC7828203

[46] Yao Q , Li W, Yu S, et al. Multifunctional chitosan/polyvinyl pyrrolidone/45S5 bioglass^®^ scaffolds for MC3T3-E1 cell stimulation and drug release. Mater Sci Eng C Mater Biol Appl. 2015;56:473-480. 10.1016/j.msec.2015.06.046 26249617

[47] Jin J , Nolte PA. Mitochondrial distribution and osteocyte mechanosensitivity. Curr Osteoporos Rep. 2025;23:22. 10.1007/s11914-025-00918-1 40402395 PMC12098195

[48] Korenkova O , Pepe A, Zurzolo C. Fine intercellular connections in development: TNTs, cytonemes, or intercellular bridges? Cell Stress. 2020;4:30-43. 10.15698/cst2020.02.212 32043076 PMC6997949

[49] Ma L , Yang X, Huang X, et al. Dynamic three-dimensional culture enhances tunneling nanotubes-mediated mitochondrial transfer in mesenchymal stromal cells to accelerate wound healing. J Nanobiotechnology. 2025;23:559. 10.1186/s12951-025-03655-w 40790215 PMC12337429

[50] Duncan RL , Turner CH. Mechanotransduction and the functional response of bone to mechanical strain. Calcif Tissue Int. 1995;57:344-358. 10.1007/bf00302070 8564797

[51] Mohanraj B , Duan G, Peredo A, et al. Mechanically activated microcapsules for “on‐demand” drug delivery in dynamically loaded musculoskeletal tissues. Adv Funct Mater. 2019;29:1807909. 10.1002/adfm.201807909 32655335 PMC7351315

[52] Youlten SE , Kemp JP, Logan JG, et al. Osteocyte transcriptome mapping identifies a molecular landscape controlling skeletal homeostasis and susceptibility to skeletal disease. Nat Commun. 2021;12:2444. 10.1038/s41467-021-22517-1 33953184 PMC8100170

[53] McNamara LM. Osteocytes and estrogen deficiency. Curr Osteoporos Rep. 2021;19:592-603. 10.1007/s11914-021-00702-x 34826091

[54] Lewis KJ , Cabahug-Zuckerman P, Boorman-Padgett JF, et al. Estrogen depletion on in vivo osteocyte calcium signaling responses to mechanical loading. Bone. 2021;152:116072. 10.1016/j.bone.2021.116072 34171514 PMC8316427

[55] Anastasilakis A , Polyzos S, Makras P, et al. Circulating irisin is associated with osteoporotic fractures in postmenopausal women with low bone mass but is not affected by either teriparatide or denosumab treatment for 3 months. Osteoporos Int. 2014;25:1633-1642. 10.1007/s00198-014-2673-x 24599275

[56] Rauner M , Taipaleenmäki H, Tsourdi E, Winter EM. Osteoporosis treatment with anti-sclerostin antibodies - mechanisms of action and clinical application. J Clin Med. 2021;10:787. 10.3390/jcm10040787 33669283 PMC7920044

[57] Kim BJ , Bae SJ, Lee SY, et al. TNF-α mediates the stimulation of sclerostin expression in an estrogen-deficient condition. Biochem Biophys Res Commun. 2012;424:170-175. 10.1016/j.bbrc.2012.06.100 22735261

[58] Iolascon G , Liguori S, Paoletta M, Toro G, Moretti A. Anti-sclerostin antibodies: a new frontier in fragility fractures treatment. Ther Adv Musculoskelet Dis. 2023;15:1759720X231197094-1759720X231197011. 10.1177/1759720x231197094 PMC10492476 37694185

[59] Chan CY , Subramaniam S, Mohamed N, et al. Circulating biomarkers related to osteocyte and calcium homeostasis between postmenopausal women with and without osteoporosis. Endocr Metab Immune Disord Drug Targets. 2021;21:2273-2280. 10.2174/1871530321666210809154456 34370656

[60] Yokoyama Y , Kameo Y, Kamioka H, Adachi T. High-resolution image-based simulation reveals membrane strain concentration on osteocyte processes caused by tethering elements. Biomech Model Mechanobiol. 2021;20:2353-2360. 10.1007/s10237-021-01511-y 34471950 PMC8595188

[61] Klein-Nulend J , Bacabac RG, Bakker AD. Mechanical loading and how it affects bone cells: the role of the osteocyte cytoskeleton in maintaining our skeleton. Eur Cell Mater. 2012;24:278-291. 10.22203/ecm.v024a20 23007912

[62] Vatsa A , Breuls RG, Semeins CM, Salmon PL, Smit TH, Klein-Nulend J. Osteocyte morphology in fibula and calvaria—is there a role for mechanosensing? Bone. 2008;43:452-458. 10.1016/j.bone.2008.01.030 18625577

[63] Jin J , Jaspers RT, Wu G, Korfage JAM, Klein-Nulend J, Bakker AD. Shear stress modulates osteoblast cell and nucleus morphology and volume. Int J Mol Sci. 2020;21:8361. 10.3390/ijms21218361 33171812 PMC7664694

[64] Bacabac RG , Mizuno D, Schmidt CF, et al. Round versus flat: bone cell morphology, elasticity, and mechanosensing. J Biomech. 2008;41:1590-1598. 10.1016/j.jbiomech.2008.01.031 18402963

[65] Wu V , Van Oers RFM, Schulten EAJM, Helder MN, Bacabac RG, Klein-Nulend J. Osteocyte morphology and orientation in relation to strain in the jaw bone. Int J Oral Sci. 2018;10:2. 10.1038/s41368-017-0007-5 29483534 PMC5944599

[66] Chen Y , Klein-Nulend J, Bravenboer N. Ageing-related changes in ultrastructural bone matrix composition and osteocyte mechanosensitivity. Curr Osteoporos Rep. 2025;23:35. 10.1007/s11914-025-00927-0. 40794153 PMC12343736

[67] Niu X , Fan R, Guo X, et al. Shear-mediated orientational mineralization of bone apatite on collagen fibrils. J Mater Chem B. 2017;5:9141-9147. 10.1039/c7tb02223a 32264595

[68] Van Hove RP , Nolte PA, Vatsa A, et al. Osteocyte morphology in human tibiae of different bone pathologies with different bone mineral density—is there a role for mechanosensing? Bone. 2009;45:321-329. 10.1016/j.bone.2009.04.238 19398046

[69] Ferretti M , Palumbo C. Static osteogenesis versus dynamic osteogenesis: a comparison between two different types of bone formation. Appl Sci. 2021;11:2025. 10.3390/app11052025

[70] Wu V , Klein-Nulend J, Bravenboer N, Ten Bruggenkate CM, Helder MN, Schulten EAJM. Long-term safety of bone regeneration using autologous stromal vascular fraction and calcium phosphate ceramics: a 10-year prospective cohort study. Stem Cells Transl Med. 2023;12:617-630. 10.1093/stcltm/szad045 37527504 PMC10502529

[71] Prins HJ , Schulten EAJM, Ten Bruggenkate CM, Klein-Nulend J, Helder MN. Bone regeneration using the freshly isolated autologous stromal vascular fraction of adipose tissue in combination with calcium phosphate ceramics. Stem Cells Transl Med. 2016;5:1362-1374. 10.5966/sctm.2015-0369 27388241 PMC5031181

[72] Vanvelk N , de Mesy Bentley KL, Verhofstad MH, Metsemakers WJ, Moriarty TF, Siverino C. Development of an ex vivo model to study *Staphylococcus aureus* invasion of the osteocyte lacuno‐canalicular network. J Orthop Res. 2025;43:446-456. 10.1002/jor.25988 39380444

[73] Seddiqi H , Abbasi-Ravasjani S, Saatchi A, et al. Osteogenic activity on NaOH-etched three-dimensional-printed poly-ɛ-caprolactone scaffolds in perfusion or spinner flask bioreactor. Tissue Eng Part C Methods. 2023;29:230-241. 10.1089/ten.tec.2023.0062 37253166

[74] Seddiqi H , Saatchi A, Amoabediny G, et al. Inlet flow rate of perfusion bioreactors affects fluid flow dynamics, but not oxygen concentration in 3D-printed scaffolds for bone tissue engineering: Computational analysis and experimental validation. Comput Biol Med. 2020;124:103826. 10.1016/j.compbiomed.2020.103826 32798924

[75] Klein-Nulend J , Semeins CM, Ajubi N, Nijweide PJ, Burger EH. Pulsating fluid flow increases nitric oxide (NO) synthesis by osteocytes but not periosteal fibroblasts-correlation with prostaglandin upregulation. Biochem Biophys Res Commun. 1995;217:640-648. 10.1006/bbrc.1995.2822 7503746

[76] Juffer P , Bakker AD, Klein-Nulend J, Jaspers RT. Mechanical loading by fluid shear stress of myotube glycocalyx stimulates growth factor expression and nitric oxide production. Cell Biochem Biophys. 2014;69:411-419. 10.1007/s12013-013-9812-4 24402674

[77] Jin J , Seddiqi H, Bakker AD, et al. Pulsating fluid flow affects pre‐osteoblast behavior and osteogenic differentiation through production of soluble factors. Physiol Rep. 2021;9:e14917. 10.14814/phy2.14917 34174021 PMC8234477

[78] Zhang J , Griesbach J, Ganeyev M, et al. Long-term mechanical loading is required for the formation of 3D bioprinted functional osteocyte bone organoids. Biofabrication. 2022;14:035018. 10.1088/1758-5090/ac73b9 35617929

[79] Mollentze J , Durandt C, Pepper MS. An in vitro and in vivo comparison of osteogenic differentiation of human mesenchymal stromal/stem cells. Stem Cells Int. 2021;2021:9919361. 10.1155/2021/9919361 34539793 PMC8443361

[80] Liu H , Chen H, Han Q, et al. Recent advancement in vascularized tissue-engineered bone based on materials design and modification. Mater Today Bio. 2023;23:100858. 10.1016/j.mtbio.2023.100858 PMC10679779 38024843

